# Recurrent extraneural sonic hedgehog medulloblastoma exhibiting sustained response to vismodegib and temozolomide monotherapies and inter-metastatic molecular heterogeneity at progression

**DOI:** 10.18632/oncotarget.23699

**Published:** 2018-01-03

**Authors:** Gregorio J. Petrirena, Julien Masliah-Planchon, Quentin Sala, Bertrand Pourroy, Didier Frappaz, Emeline Tabouret, Thomas Graillon, Jean-Claude Gentet, Olivier Delattre, Olivier Chinot, Laetitia Padovani

**Affiliations:** ^1^ Service de Neuro-Oncologie, Hôpital La Timone, Assistance Publique-Hôpitaux de Marseille, Marseille, France; ^2^ Unité de Génétique Somatique, Département de Génétique Oncologique, Institut Curie, Paris, France; ^3^ INSERM_U830, Institut Curie, Paris, France; ^4^ Service de Médecine Nucléaire, Hôpital Nord, Assistance Publique-Hôpitaux de Marseille, Marseille, France; ^5^ Unité Oncopharma, Service Pharmacie, Hôpital La Timone, Assistance Publique-Hôpitaux de Marseille, Marseille, France; ^6^ Service de Neuro-Oncologie Pédiatrique et Adulte, Centre Léon Bérard, Lyon, France; ^7^ Aix-Marseille Université, Marseille, France; ^8^ Service de Neurochirurgie, Hôpital La Timone, Assistance Publique-Hôpitaux de Marseille, Marseille, France; ^9^ Service d’Oncologie et Hématologie Pédiatrique, Hôpital La Timone, Assistance Publique-Hôpitaux de Marseille, Marseille, France; ^10^ Service de Radiothérapie, Hôpital La Timone, Assistance Publique-Hôpitaux de Marseille, Marseille, France

**Keywords:** sonic hedgehog medulloblastoma, vismodegib, temozolomide, smoothened mutation, PIK3CA mutation

## Abstract

**Background:**

Response to targeting and non-targeting agents is variable and molecular information remains poorly described in patients with recurrent sonic-hedgehog-driven medulloblastoma (SHH-MB).

**Materials and Methods:**

Clinical and PET/CT findings during treatment with successive hedgehog antagonists and temozolomide monotherapies are described in a heavily pre-treated patient with recurrent extraneural metastases from *PTCH1* mutated/ wild type smoothened (*SMO*) CNS SHH-MB. Molecular tests were prospectively performed in tissue from two extraneural sites at progression.

**Results:**

Sustained clinical/metabolic response was obtained to vi*smo*degib. At progression, itraconazole was ineffective, but salvage temozolomide treatment results in a response similar to vismodegib. At further progression, acquired *SMO* and *PIK3CA* mutations were identified in bone (G477L and H1047A, respectively) and epidural (L412P and H1065L, respectively) metastases. No response was observed with subsequent sonidegib treatment.

**Conclusions:**

This is the first clinical report of recurrent extraneural *PTCH1* mutated SHH-MB exhibiting: 1) a sustained response to vismodegib and temozolomide, and 2) inter-metastatic molecular heterogeneity and acquired SMO-G477L, SMO-L412P, and PIK3CA-H1065L mutations at progression, highlighting the need for a multitarget treatment approach.

## INTRODUCTION

Medulloblastoma (MB) is the most common malignant pediatric brain tumor, with at least four major molecular subgroups identified. Sonic-hedgehog (SHH) subgroup MB (SHH-MB) comprises the majority of infant and adult MBs [1]. For patients with recurrent SHH-MB, survival is dismal [2]. Although disease recurrence is usually confined within the CNS, extraneural metastases may rarely develop [3–5].

Systemic SHH inhibitors, such as vismodegib and sonidegib, represent a therapeutic option for recurrent SHH-MB [6]; however, variable response rates have been reported [2], mostly due to the molecular heterogeneity among SHH-MBs [7]. Moreover, although the proof of principle of the *SMO* mutation conferring acquired resistance to vismodegib was provided in a SHH-MB patient, molecular aberrations in relapsing SHH-MB remain poorly described in the clinic setting compared to preclinical models [2, 8–10]. However, the limited impact of molecular profiling on the current management of advanced disease, makes performing invasive procedures required for tumor sampling and molecular testing difficult.

The aim of this case report was to describe: 1) the clinical/metabolic response to vismodegib and salvage treatment with temozolomide and other SHH antagonists in a patient with recurrent extraneural SHH-MB, and 2) the molecular profiling of the primary CNS tumor and extraneural metastases at disease progression.

## CASE REPORT

**Figure 1:**
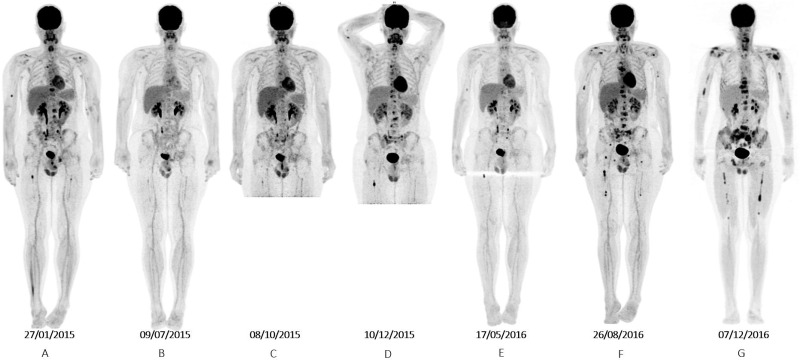
Whole body projections from F^18^–fluorodeoxyglucose PET scans showing (**A**) baseline examination before vismodegib, (**B**) partial response to vismodegib, (**C**) disease progression under vismodegib, (**D**) further progression under itraconazole, (**E**) partial response to temozolomide, (**F**) progression under temozolomide, and (**G**) progression after sonidegib with response in 6th thoracic and 3rd lumbar locally treated lesions.

**Figure 2:**
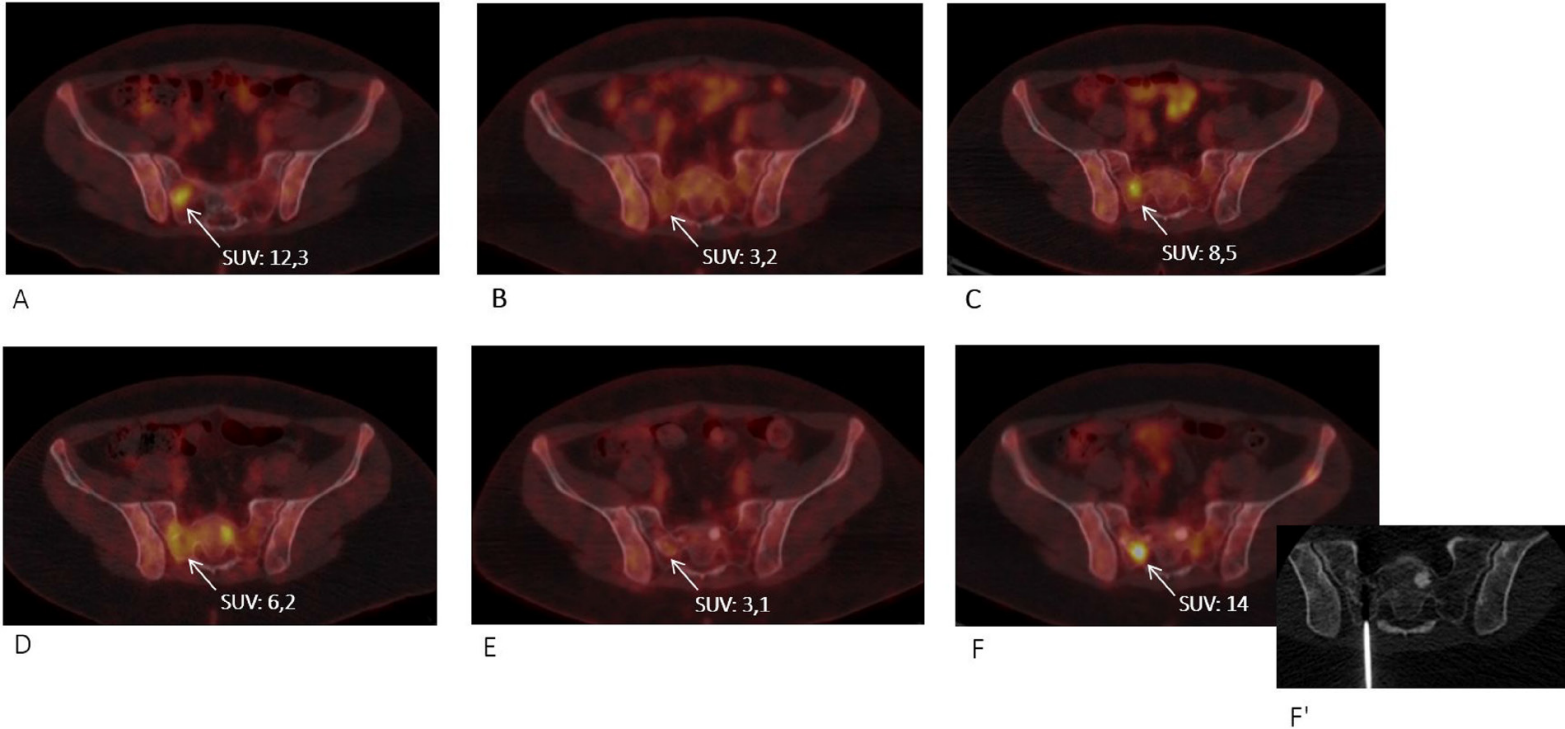
Axial F^18^–fluorodeoxyglucose PET/CT images at the pelvic level showing the right sacral alae lesion (arrow) chosen as a target for molecular analyses after progression to temozolomide (**A**) Before vismodegib monotherapy, (**B**) partial response to vismodegib, (**C**) disease progression under vismodegib, (**D**) further lesion size progression under itraconazole, (**E**) partial response to temozolomide, and (**F**) progression under temozolomide when the CT scan-guided biopsy was performed (F’).

**Figure 3:**
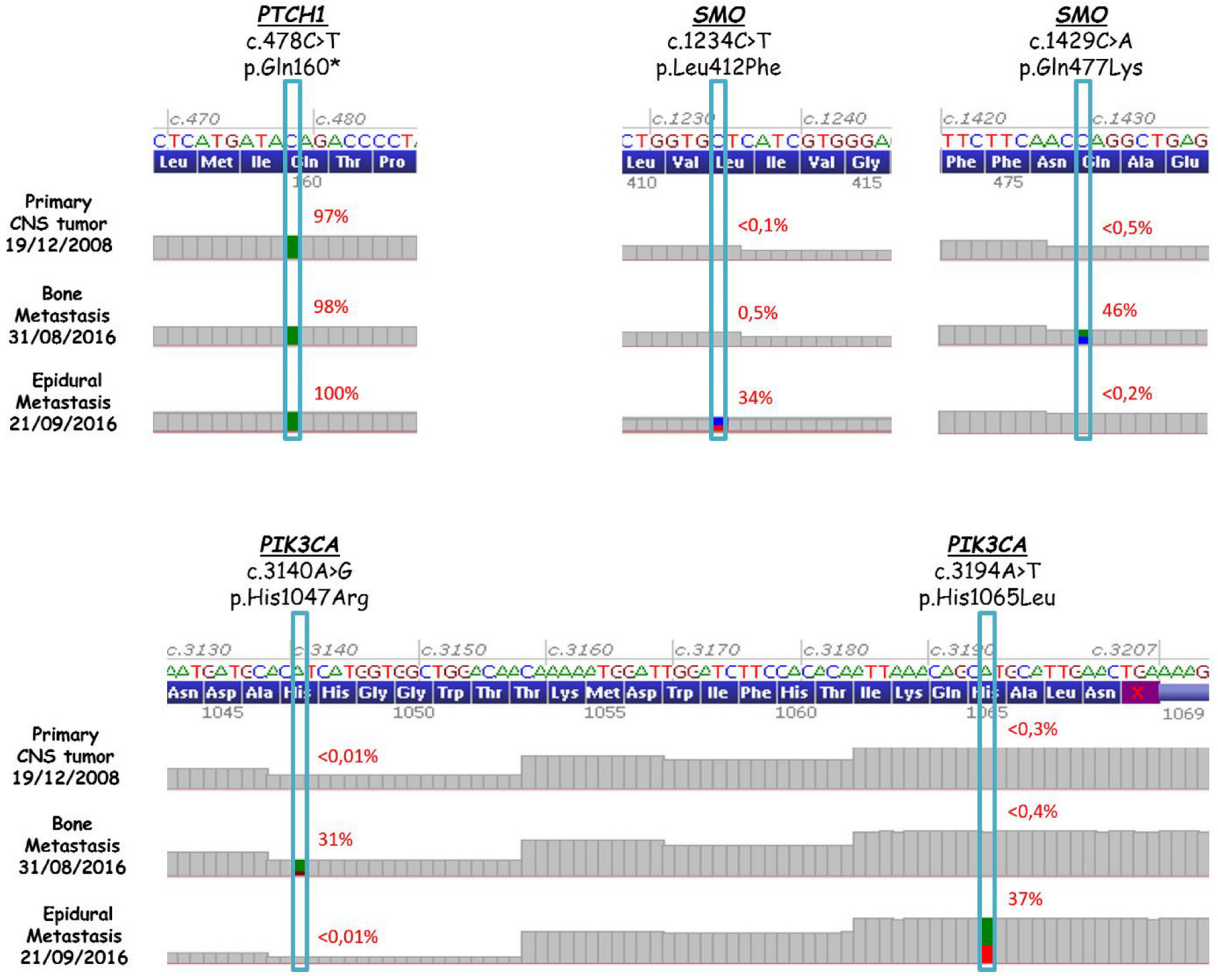
*PTCH1* (top-left), *SMO* (top-right), and *PIK3CA* (bottom) sequence analysis in the primary CNS tumor and two extraneural metastases after progression to vismodegib and temozolomide

**Figure 4:**
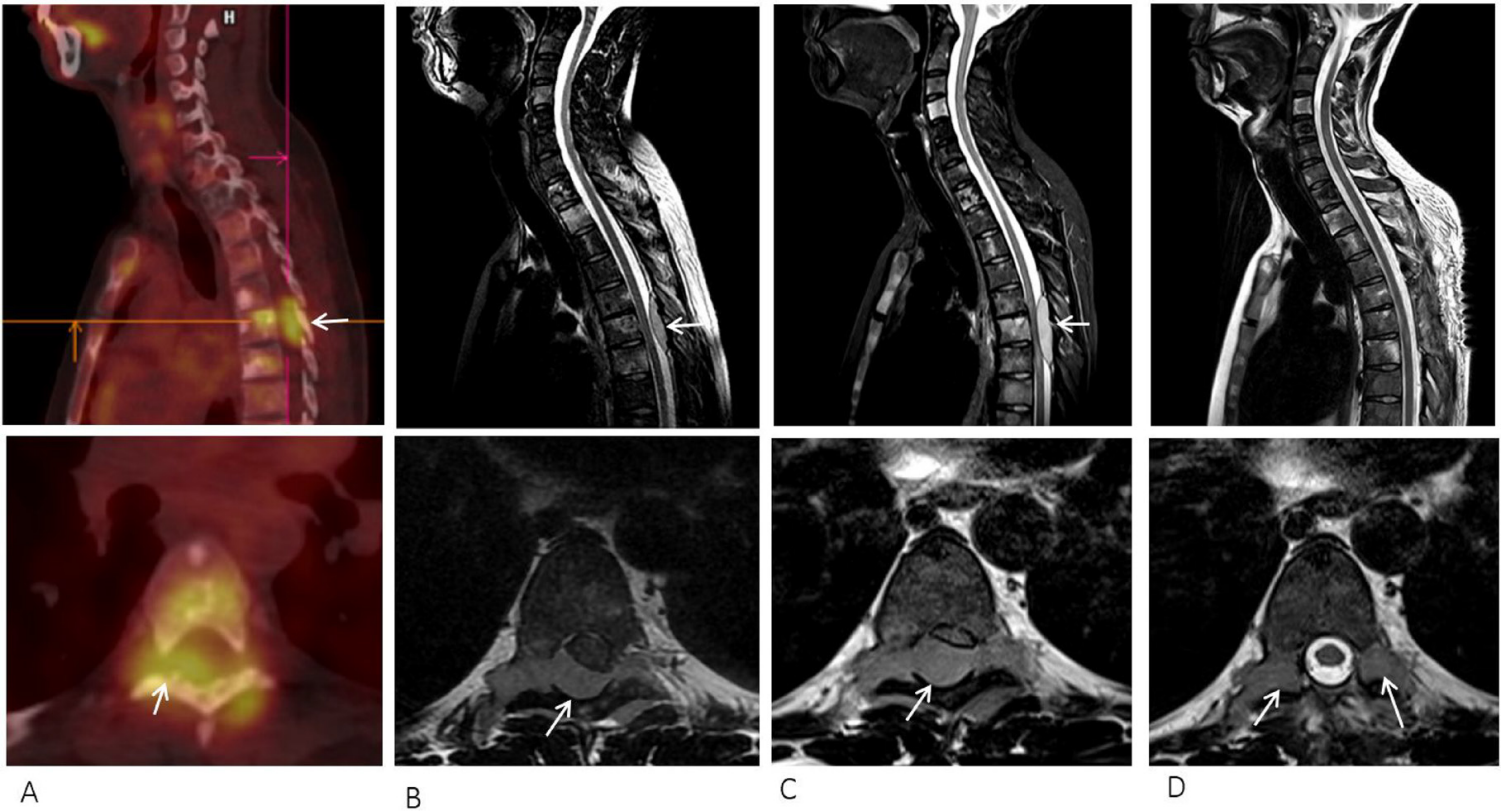
(**A**) Thoracic epidural metastasis (arrow) detected on PET/CT at progression under temozolomide. T2-weighted MRI scan of the spine at (**B**) baseline, (**C**) ten days after starting sonidegib showing tumor progression, and (**D**) after surgical decompression (raw sagittal and axial images on top and bottom, respectively).

A 16-year-old male was diagnosed with standard risk desmoplastic MB of the cerebellum in December 2008. He received bi-fractionated craniospinal irradiation (CSI; 36 Gy CSI in 36 fractions, with 68 Gy for boost on tumor bed in 68 fractions) according to the MSFOP 98 trial [11] resulting in complete response. For the isolated extraneural metastases of bone and bone marrow that occurred in November 2011, he received chemotherapy with etoposide, carboplatin, cisplatin, cyclophosphamide, irinotecan, and temozolomide, followed by intensive chemotherapy with busulfan/thiotepa and autologous stem cell transplantation. Maintenance with etoposide, celecoxib, cyclophosphamide, and temozolomide was delivered until December 2012, when a PET/CT and bone marrow examination showed complete response. In August 2014, left hip pain prompted a PET/CT scan that revealed a local hypermetabolic lesion, identified as necrotic tumor recurrence by biopsy. Focal radiotherapy with concomitant oral temozolomide allowed complete hip pain control. In January 2015, multifocal pain developed and a PET/CT scan revealed multiple hypermetabolic skeletal lesions of the spine, sternum, pelvis, and proximal extremities (Figures 1A and 2A). Retrospective immunohistochemical analysis of the primary CNS tumor showed the SHH-MB immunophenotype [12], which was further confirmed by retrospective CGH array and targeted next-generation sequencing showing chromosome 9q copy neutral-loss of heterozygosity and the *PTCH1* mutation, respectively (Table 1, Figure 3). On 28 January, 2015, he was enrolled onto the NCT01601184 study and 150 mg PO once daily vismodegib monotherapy was started. His diffuse pain disappeared three weeks after treatment initiation; hence, morphine was discontinued. Whole body PET/CT scans performed in March and May 2015 revealed a partial metabolic response (data not shown), which was further confirmed in July 2015 (Figures 1B and 2B). Grade 1 cramp/alopecia/dysgeusia and grade 2 diarrhea were observed under vismodegib without impacting his daily living. In October 2015, the back pain reappeared; a PET/CT scan revealed recurrent disease (Figures 1C and 2C), which was treated with 150 mg twice daily itraconazole. The patient had a normal itraconazole serum level (1673 ng/ml; antifungal therapeutic range, 1000–4000 ng/ml) on day 14 of treatment initiation. However, he had progressively increasing skeletal pain and serum C-reactive protein (CRP) levels. The PET/CT scan performed five weeks after treatment initiation showed skeletal disease progression (Figures 1D and 2D). He received temozolomide (first cycle 150 mg/m^2^/day then 200 mg/m^2^/day) five days per month. Three weeks after the second cycle, pain began to improve and the elevated serum CRP level diminished. A PET/CT scan performed after four cycles of temozolomide showed a partial metabolic response while the patient was asymptomatic with almost normal serum CRP levels (Figures 1E and 2E). He remained asymptomatic and monthly temozolomide was continued until August 2016 when progressive multifocal pain reappeared and his CRP level increased. Multifocal skeletal recurrence was observed on a PET/CT scan (Figures 1F and 2F) and the thoracic paravertebral hypermetabolic foci (Figure 4A) were confirmed as an epidural metastasis on MRI (Figure 4B). A sacral biopsy was performed for molecular analysis after obtaining the patient’s consent (Figure 2F). On September 9, 2016, 400 mg (oral suspension) once daily sonidegib was started. Ten days later he rapidly developed numbness in the lower trunk and extremities, paraparesis, and urinary retention requiring urgent neurosurgical evaluation. MRI showed thoracic epidural mass progression with increased spinal cord compression (Figure 4C), as well as a new epidural lesion at the 3rd lumbar vertebral level with incipient cauda equina compression (data not shown). Sonidegib was temporarily discontinued and emergency surgery performed, resulting in immediate neurological improvement and successful spinal cord decompression (Figure 4D). On postoperative Day 4, 800 mg once daily Sonidegib was restarted. Focal radiotherapy consisting three fractions of 6 Gy and three fractions of 8 Gy was delivered every two days on the postoperative thoracic and lumbar extradural lesions, respectively. Pain temporarily improved and his CRP level almost normalized; however, on November 2, 2016, sonidegib was interrupted due to grade 4 serum creatine kinase (CK) elevation. While 400 mg once daily sonidegib was restarted after CK normalization, vomiting precluded a good adherence thereafter. The patient experienced severe multifocal pain and increased serum CRP levels within three weeks and in December 2016, a PET/CT scan showed new extraneural lesions within and outside the skeleton. Comparative molecular analysis between the primary CNS tumor and relapsing bone and epidural metastases showed a similar *PTCH1* mutated profile. However, acquired *SMO* (G477L and L412P) and *PIK3CA* (H1047A and H1065L) mutations with inter-tumoral heterogeneity were identified without evidence of SHH activation downstream of SMO or *TP53* and *MYC*/*MYCN* aberrations (Table 1, Figure 3). The patient died of progressive disease four months later despite a new salvage regimen.

**Table 1:** Molecular analyses of primary CNS tumor and two extraneural metastases after progression to vismodegib and temozolomide monotherapies

	*PTCH 1*	*SMO*	*SUFU*	*PIK3CA*	*TP53*	*GLI 2*	*MYC/MYCN*	*MGMT* promoter
Primary CNS tumor (19/12/2008)	p.Gln160^*^ hmz mut	wt	wt	wt	wt	n-amp	n-amp/n-amp	unmethylated
Bone metastasis (31/08/2016)	p.Gln160^*^ hmz mut	p.Gln477Lys mut	wt	p.His1047Arg mut	wt	n-amp	n-amp/n-amp	NA
Epidural metastasis (21/09/2016)	p.Gln160^*^ hmz mut	p.Leu412Phe mut	wt	p.His1065Leu mut	wt	n-amp	n-amp/n-amp	unmethylated

Abbreviations: hmz, hemizygous; mut, mutation; wt, wild type; n-amp, non-amplified; NA, not available.

## DISCUSSION

This case illustrates that recurrent extraneural metastatic SHH-MB is not an inevitable end-stage condition, and that even heavily pre-treated patients should be considered for targeted and non-targeted treatments.

Although molecular subtype data was not available in the largest reported series of extraneural metastatic MB [5], the correlation between desmoplastic histology and SHH activation [12] suggests that the majority of long-term survivors belong to the SHH-MB subgroup [5]. Similar to our case, most of desmoplastic MB patients only received CSI as the initial treatment, raising the question of the validity of systematic chemotherapy to eradicate subclinical metastatic extraneural disease in this MB subgroup.

Sustained objective response with an acceptable toxicity profile to vismodegib was observed in our case with *PTCH1* mutation-driven SHH-MB, supporting the idea that isolated mutations upstream of SMO without downstream activating aberrations predict the response to SMO inhibitors [7]. Similarly, in the largest clinical trial of SMO inhibitors for recurrent SHH-MB, somatic loss-of-heterozygosity of *PTCH1* was associated with prolonged progression-free survival, and loss-of-function *PTCH1* mutations were only found among responders [2]. The role of vismodegib as a maintenance therapy after traditional chemotherapy for newly diagnosed SHH-MB is currently under investigation (NCT01878617).

It is important to note that, in our case, tumor profiling was unknown at the time of vismodegib initiation. However, the sustained response to vismodegib strongly suggests the maintenance of the homozygous *PTCH1* mutation as the predominant oncodriver at this time. The presumed genomic stability during the first six years of multitreated disease could be partly related to the lack of *TP53* mutation in the primary tumor [13, 14], which is otherwise consistent with the prolonged survival of our patient. In contrast, a recent publication showed a poor overlap of genetic events in recurrent murine SHH-MB after “humanized” *in vivo* therapy with those in matched murine diagnostic samples [15]. Similarly, substantial genetic divergence was observed between human diagnostic and recurrent MB after standard therapy. Interestingly, one SHH-MB patient with a clonally dominant homozygous *PTCH1* driver mutation in the primary tumor exhibited a complete switch in the oncodriver mutation in the recurrent tumor (derived from ancestral lineage with wild-type chro9q heterozygosity). Thus, the authors emphasized the importance of performing rebiopsies and profiling the recurrent compartments to more appropriately direct subsequent therapies [15].

Unfortunately, acquired resistance to SMO antagonists is uniformly reported in responder SHH-MB patients [2, 4], as illustrated in our case. In accordance with a recent study [16], the two metastatic compartments examined in our case retained the same molecular affiliation as the primary CNS tumor; however, acquired mutations were also found in both sites with an inter-tumoral molecular heterogeneity profile.

The spectrum of mutant *SMO* conferring SHH inhibitor resistance has rapidly expanded in recent years, after the first mutation, D473H, was identified in a metastatic SHH-MB patient exhibiting a dramatic but only transient response to vismodegib [3, 8]. Recent clinical studies in basal cell carcinoma (BCC), a frequent SHH-driven tumor, showed that *SMO* genetic alterations include ligand binding pocket (LBP) mutations, which define sites of inhibitor binding, as well as mutations in structural pivot regions conferring constitutive receptor activity and drug resistance. These topographically different *SMO* mutations may confer both intrinsic and acquired resistance to SHH inhibitors [17, 18].

The SMO-G477L mutation identified in the relapsing sacral bone metastasis of our patient was recently described among the SMO-LBP mutants in vismodegib-resistant BCC [17]. Atwood et al. found that this mutant exhibited a high level of drug resistance (the vismodegib half-maximal inhibitory concentration [IC_50_] was 40-fold higher compared with *SMO* wild type), without significantly altering basal SHH pathway activity. Progression of the sacral lesion under vismodegib after a transient partial metabolic response (Figure 2) suggests that expansion of SMO-G477L mutant subclones most likely occurred during vismodegib therapy.

The SMO-L412P mutation was identified in the epidural metastases detected after progression under temozolomide. This mutation which is located outside the LBP of SMO and previously reported as an oncogenic driver, was recently identified in pre- and post-treatment samples of a patient with metastatic BCC who initially responded to vismodegib. The authors speculated that the acquired loss of the *PTCH1* mutation in the setting of this oncogenic mutation probably promoted tumor regrowth under vismodegib in their patient [18].

Hyperactivation of phosphatidylinositol 3-kinase (PI3K) signaling cascades is one of the most common events in human cancers, often via *PIK3CA* hotspot missense mutations [19]. Recurrent *PIK3CA*, *PTEN,* and *PIK3C2G* mutations were identified in the largest genome sequencing series of newly diagnosed SHH-MB patients; the vast majority of lesions with immunostaining evidence of PI3K pathway activation were from adult patients, and immunostaining positivity was strongly associated with poor outcome in this population [7]. Moreover, Robinson et al. also identified *PIK3* and *PTEN* mutations in the SHH-MB subset that did not respond to vismodegib [2].

In the present case, however, no evidence of PI3K pathway activation was observed in the primary CNS tumor. Instead, *PIK3CA* mutations were identified in the setting of advanced extraneural relapsing disease, suggesting a potential role in MB progression rather than in tumor initiation, as suggested by preclinical models [20]. The H1047A mutation, which is the most frequent activating aberration of the *PIK3CA* gene encoding the p110α catalytic subunit (exon 20) of the class I PI3Ks in human tumors, was identified in the sacral bone metastasis. Interestingly, the mutation found in the epidural metastasis at 1065 amino acid position of exon 20 has not been previously described in human SHH-MB. This mutation considered infrequent but with a high pathogenic score according to the COSMIC database (http://www.sanger.ac.uk/genetics/CGP/cosmic), has been reported in deeply invasive endometrial carcinoma and salivary gland tumors, and invasive lobular breast cancer [21]. Additional data suggest that this variation could be pathogenic: both the nucleotide (c.3194) and the amino acid (p.1065) are highly conserved, the biochemical distance between histidine and leucine is significant, and this variation is located in the catalytic domain of the protein.

The precise mechanism by which *PIK3CA* and *SMO* mutations interplayed to promote tumor progression in our patient is unknown. Previously, Riobó et al. demonstrated in several experimental systems that activation of PI3K/Akt increases sonic-hedgehog-induced GLI transcriptional activity by inhibiting PKA-dependent GLI2 inactivation [22]. Cumulative data indicate that SMO-independent hedgehog signaling, namely non-canonical hedgehog signaling, plays an essential role in cancer through activation of GLI as the output for numerous other oncogenic pathway [23]. In line with this, dual PI3K/mTOR inhibition was recently found to exert potent antineoplastic effects and to decrease nuclear localization of GLI, as well as GLI target gene expression in DAOY and D556 MB cell lines [24]. Accordingly, restored canonical SHH pathway via *SMO* mutations leading to the evasion of SMO antagonists probably synergized with enhanced PI3K signaling to promote GLI activation in our case. However, it is speculative and alternative pathway crosstalk leading to disease progression cannot be excluded.

Objective sustained response was also observed to temozolomide. Similar results have been reported for both extraneural and CNS relapsing MB; however, the molecular subtyping was not provided [25–27]. To our knowledge, the present case is the first report of successful temozolomide salvage treatment after vismodegib progression, suggesting that acquired resistance to vismodegib does not necessarily promote the development of an alkylating resistance phenotype. Of note, the objective therapeutic response was observed despite an unmethylated *MGMT* promoter. This is in accordance with a recent publication showing that the expression of MGMT is not predictive of intrinsic alkylating agent resistance in MB [28]. Whether a more prolonged response was possible with combined vismodegib and temozolomide therapy rather than monotherapies in our patient is unclear; the ongoing trial (NCT01601184) is addressing this question.

At the time of progression to vismodegib, we have attempted hedgehog pathway inhibition differently. In a mouse allograft model, itraconazole inhibited SHH pathway activity and MB growth at serum levels comparable to those in patients undergoing antifungal therapy, presumably by binding to a site distinct from that of cyclopamine [29]. The authors subsequently showed that itraconazole prolonged the survival of mice with intracranial vismodegib-resistant tumors harboring D477G mice mutation, homologous to the D473H human mutation [30]. However, no therapeutic response was observed in our patient despite adequate serum levels, possibly because itraconazole acts as a partial SMO mutant antagonist, with higher doses being required to overcome the elevated IC_50_ and resistance in human vismodegib-induced SMO mutants. It might also reflect limitations of preclinical models in reproducing real clinical scenarios and/or that alternative oncodrivers, such as PIK3 pathway activation, were already present. To date, no clinical experience concerning the use of itraconazole in vismodegib-naïve or resistant SHH-MB has been reported. In a recent exploratory phase II trial, itraconazole showed anti-BCC activity in vismodegib-naïve patients. However, similar to our case, the three patients with vismodegib-resistant BCC receiving 200 mg twice daily itraconazole experienced progression during therapy [31].

In a phase I trial, sonidegib exhibited an acceptable safety profile, exposure-dependent reduction in GLI1 mRNA expression, and clinically relevant antitumor effect in patients with relapsed SHH-MB [32]. In a more recent I-II study, four patients (two children and two adults) among 10 with relapsing SHH-MB achieved complete response under sonidegib, but the correlation between response and hedgehog pathway alterations could not be evaluated due to insufficient patient material [33].

Nevertheless, the potential usefulness of sonidegib in SHH-MB patients with acquired resistance to vismodegib has not been previously reported. In a recent clinical study, patients with advanced BCC who developed resistant to vismodegib did not respond to sonidegib, yet one patient with a baseline SMO-D473 mutation had stable disease for 58 weeks, presumably due to intra-tumoral heterogeneity [34]. Early treatment discontinuation due to adverse events makes analysis complex in our patient. Nevertheless, severe progression three weeks after treatment interruption despite the relatively long half-life of sonidegib [32] strongly suggests limited antitumoral activity. Of note, the SMO-G477L mutation, which is known to confer resistance to sonidegib in BCC [34], and PI3K molecular aberrations were already present at the time of treatment initiation.

The main limitation of this report is the lack of molecular information at the time of each new therapeutic intervention. However, this reflects clinical settings and does not dramatically hamper, in our opinion, the relevance of our findings.

## CONCLUSIONS

Recurrent extraneural *PTCH1* mutated SHH-MB does not necessarily represent an end-stage condition. Vismodegib and temozolomide monotherapies allowed sustained responses with good quality daily living in our heavily pre-treated patient. However, salvage therapy with other SHH inhibitors after acquired resistance to vismodegib did not provide clear benefit.

Inter-metastatic molecular heterogeneity observed at disease progression suggests that spatially variable subclone selection occurred during treatment. SMO-G477L, SMO-L412P, and PIK3CA-H1065L mutations are identified for the first time in a recurrent SHH-MB patient. These features and the *bona fide* oncogenic mutant PIK3CA-H1047A identified in our patient highlight the cooperating role of SHH and PIK3 pathways in the development of acquired resistance and disease progression in SHH-MB. Therefore, as supported by preclinical studies [10] multitarget therapy may be a more adequate strategy to treat this aggressive cancer.

## Abbreviations

MB: medulloblastoma
SHH: sonic-hedgehog signaling pathway
SHH-MB: sonic hedgehog-driven medulloblastoma
*PTCH1*: gene encoding patched homologue 1, PTCH1
*SMO*: gene encoding smoothened receptor, SMO
PET/CT: positron emission tomography/computed tomography
CNS: central nervous system
CSI: craniospinal irradiation
CRP: serum C-reactive protein
MRI: magnetic resonance imaging
CK: creatine kinase
*MGMT*: gene encoding methylguanine methyltransferase, MGMT
BCC: basal cell carcinoma
LBP: ligand binding pocket
IC_50_: half-maximal inhibitory concentration
PIK3: phosphatidylinositol 3-kinase
COSMIC: Catalogue of Somatic Mutations in Cancer
